# Comparison of QT Dispersion With Left Ventricular Mass Index in Early Diagnosis of Cardiac Dysfunction in Patients With β-Thalassemia Major

**DOI:** 10.5812/ircmj.11698

**Published:** 2014-05-05

**Authors:** Noor Mohammad Noori, Maziar Mahjoubifard, Mehdi Mohammadi, Alireza Jahangiri Fard, Abdolhossein Abassi, Behrooz Farzanegan

**Affiliations:** 1 Children and Adolescents’ Health Research Center, Zahedan University of Medical Sciences, Zahedan, IR Iran; 2 Tracheal Diseases Research Center, Shahid Beheshti University of Medical Sciences, Tehran, IR Iran; 3 Zahedan University of Medical Sciences, Zahedan, IR Iran

**Keywords:** Beta-Thalassemia, Cardiac involvement, Electrocardiography, Echocardiography

## Abstract

**Background:**

In electrocardiography (ECG), QT is the interval between the onset of Q wave to the end of the T wave. This interval may be a sign of changes in the ventricular structure in hematologic disorders such as thalassemia major.

**Objectives:**

The main goal of this study was to compare the diagnostic value of corrected QT dispersion (QTcd) and QT dispersion (QTd) with left ventricular mass (LVM) and left ventricular mass index (LVMI) as well as to determine their sensitivity and specificity in early detection of the cardiac involvement in patients with β-thalassemia major.

**Patients and Methods:**

In a case-control study, 60 patients older than ten years of age with thalassemia major who received regular blood transfusion and iron chelators were selected as the case group and were compared with 60 healthy age- and sex-matched subjects. All patients had myocardial performance index (MPI) of more than 0.5 and MPI for controls was less than 0.5. Echocardiography and ECG were performed for both groups and data were analyzed using appropriate statistical tests.

**Results:**

The mean age of cases and controls were 16 ± 2.8 and 16.08 ± 3.01 years, respectively. Male to female ratio was 33:27 in case group and 31:29 in the control group. LVMI in the case group was greater than control group. QTd and QTcd were larger in case group than in control group. The sensitivity and specificity of LVM, LVMI, QTd, and QTcd were as follows: 88.3%, 77.1%; 86.7%, 80%; 93.8%, 80%; and 91.7%, 86.7%, respectively.

**Conclusions:**

This study showed acceptable sensitivity and specificity of QTcd and QTd in comparison to LVMI; it seems that standard ECG can be used for early diagnosis of cardiac involvement in asymptomatic patients with thalassemia major.

## 1. Background

Thalassemia major is a common genetic disorder that causes severe anemia from early childhood. Annually 60000 newborns with thalassemia major are born worldwide (1). During the last three decades, the treatment of patients with multiple blood transfusions and systemic iron chelating agents has caused a significant improvement in the quality of life and life expectancy of these patients (2). In a study, Khosoosi Niaki et al. showed that cardiovascular diseases are the main causes of death in patients with chronic renal disease and prolong hemodialysis. Moreover, their study illustrated that in patients with renal failure, duration of QT interval, corrected QT (QTc), and QT dispersion (QTd) were increased before hemodialysis. It seems that prolongation of QT interval indices were due to potassium and calcium ions before hemodialysis, but they might not be responsible for these problems after it (3).

The durations of maximum QTc and corrected QT dispersion (QTcd) were significantly longer in pulmonary hypertension group than in the other groups. We found that the risk of atrial and ventricular arrhythmias was increased in the patients with both congenital heart disease and pulmonary hypertension (4). It seems that in addition to iron overload, myocardial fibrosis, structural changes, and ventricular hypertrophy secondary to chronic anemia lead to heart failure in these patients (5). However, the early detection and treatment of heart failure due to any reason in patients with thalassemia major improve the prognosis and increase the life expectancy. Echocardiography is a useful and noninvasive diagnostic tool for diagnosis of heart disorders and helps with early detection of heart failure before the development of symptoms. As a result, it is possible to initiate early treatment (6). It seems that electrocardiography (ECG) is a suitable alternative tool in this regard and has an acceptable test values in comparison with echocardiography (6). Based on previous studies, QTcd and QTd indices are good parameters in this regard. In a study conducted in 2006, Ulger et al. demonstrated an association between increased QTd and QTcd with echocardiographic measurements such as left ventricular mass index (LVMI) in patients with thalassemia and showed increased level of these indices in comparison with control group (6). A study conducted in 2003 by Kocharian et al. showed increased QTcd and QTd in patients with thalassemia major in comparison with control group (7). Finding of this study showed that in patients with thalassemia major and thalassemia intermedia, systolic and diastolic functions of the right and left hearts would be impaired. Therefore, serial echocardiography is recommended in asymptomatic patients with β-thalassemia for an early diagnosis of heart dysfunction (8).

Based on the previous literature, LVMI is one of the first echocardiographic indices that is increased in left ventricular hypertrophy and it is an indicator of progression to cardiac dysfunction (9, 10). As a result, the frequent and periodic echocardiographic examination is not only useful in asymptomatic patients with thalassemia major but also essential for early detection of cardiac involvement (11). The study by Noori et al. revealed that echocardiography was more accurate tool than ECG in defining the LVMI in patients with thalassemia major (12). Echocardiography is an expensive as well as specialized tool that needs advanced training and is not available in most centers (13); hence, it would be very useful to find an inexpensive as well as simple diagnostic tool for detection of cardiac involvement, which could be a sign of changes in ventricular structure in patients with thalassemia major,. However, these parameters are not specific for such patients and might be increased in other disorders such as primary hypertension, congestive heart failure (CHF), renal disease, and obesity (6).

## 2. Objectives

In the present study, we aimed to compare the diagnostic value of QTcd and QTd with LVMI as well as to determine the sensitivity and specificity of these indices in detection of cardiac involvement in patients with thalassemia major.

## 3. Patients and Methods

In a case-control study, 60 patients older than 10 years of age were randomly selected from 380 patients with thalassemia major who attended the Ali-e-Asghar Hospital, Zahedan, Iran, during 2009 and 2010 to receive regular and frequent blood transfusion. Furthermore, 60 healthy children who attended the clinic for routine examination were considered as control group. All cases had myocardial performance index (MPI) of more than 0.5 and MPI for all controls was less than 0.5. The cases and controls were matched for age and sex at the category level.

None of the patients had symptoms of heart failure at presentation; they were evaluated by a cardiologist and undergone echocardiographic examination every six months. Patients with clinical signs of CHF, arrhythmias, heart valve diseases, hypertension, obesity, metabolic, renal, or cardiac disorders, and the patients who needed complex cardiac treatments were excluded from the study. Patients were compared with 60 healthy individuals. Patients’ selection was done using a random sampling method. To assess eligibility, patients were examined carefully. It should be noted that the hemoglobin level of patients was more than 9 g/dL before blood transfusion and they had received continuous iron chelation for at least five years. After obtaining a written informed consent, all cases and controls underwent simultaneous 12-leads standard ECG and echocardiography. ECG and echocardiography were employed to measure QTcd as well as QTd and LVMI, respectively. One week after blood transfusion, ECG and echocardiography were performed in the supine position without holding the breath. The M-mode, two-dimensional, and Doppler echocardiography (Challenge 7000 with 2.5/3.5 and 3.5/5 MHz transducer, Italy) were performed by a pediatric cardiologist. Following formulas were used to calculate LVMI (Equations 1 and 2):

Equation 1.

*LVMI* = *LVM* / *Ht*^2.7^ (*g* / *m*^2.7^)

Equation 2.

*LV mass (LVM)* = 0.8 [1.04 (*IVS* + *PWT* + *LVID*)^3^.-.(*LVID*)^3^].+ 0.6 (*g*)

The 12-lead standard ECG was performed using a single device (FOKUDA, model 3500, Japan) to evaluate QT interval, QTc using the Bazett's Formula (QT/√R-R), and QTd (the difference between the minimum and maximum QT interval in at least three complexes of any lead of at least eight leads) for all participants.

### 3.1. Statistical Analysis

The data were analyzed using the SPSS version 19 (SPSS Inc., Chicago, IL, USA). Categorical data were presented as numbers and percentages and quantitative variables were presented as means ± standard deviation. As quantitative variables were not distributed in a normal pattern at least in either case or control groups based on Shapiro-Wilk test, Mann-Whitney U test was used to compare the two groups. All P values were two-tailed and P < 0.05 was considered statistically significant. Receiver operating characteristic (ROC) curve was used to determine cut-off points. In addition, blood pressure in both groups was measured with a mercury sphygmomanometer (made in japan) in the sitting position and by a cardiologist. Height (cm) and weight (kg) was measured and body mass index (BMI) was calculated by using the following formula: BMI = Weight (kg)/Height (m^2^).

The study’s protocol was approved by Research Ethics Committee in Medical Sciences of University of Zahedan. Number and date of ethical date was 90-331 and February 13, 2012. Ethical codes included 1, 3, 5, 8, 17, and 24. Informed consent was obtained from the parents before enrollment of their children in the study. 

## 4. Results

In the case group, there were 33 (55%) male and 27 (45%) female. In the control group, 31 (51.6%) patients were male and 29 (48.4%) patients were female. The mean age, BMI, systolic and diastolic blood pressures, heart rate, and hemoglobin level of participants are presented in Table 1. The mean of ejection fraction (EF), fractional shorting (FS), LVM, LVMI, isovolumic relaxation time (IVRT), peak E velocity to peak A velocity ratio (E/A), left ventricular end-diastolic dimension (LVEDD), and MPI are shown in Table 2.

**Table 1.** Demographic and Characteristic of the Case and Control Groups ^a,b^

Control	Patient	Parameter	P value
**Age, y**	16.00 ± 2.87	16.08 ± 3.01	0.962
**BMI, m** ^**2**^	18.70 ± 2.06	27.62 ± 6.92	< 0.001
**Heart Rate, beat/min**	92.28 ± 10.61	81.18 ± 10.22	< 0.001
**Hemoglobin, g/dL**	10.37 ± 0.34	14.32 ± 1.09	< 0.001
**Systolic blood pressure, mm Hg**	91.25 ± 8.11	98.18 ± 22.76	< 0.001
**Diastolic blood pressure, mm Hg**	64.66 ± 6.02	68.91 ± 6.042	< 0.001

^a^ Data are presented in mean ± SD.

^b^ Abbreviation: BMI, body mass index.

**Table 2.** Left Heart Echocardiographic Parameters in the Case and Control Groups ^a, b^

	Patient	Parameter	P value
**EF, %**	66.30 ± 6.89	67.01 ± 5.70	0.809
**FS, %**	37.08 ± 4.70	37.2 ± 4.28	0.897
**LVM, g/m** ^**2**^	102.51 ± 21.10	69.61 ± 15.96	< 0.001
**LVMI, g/m** ^**2.7**^	35.48 ± 7.85	25.25 ± 2.25	< 0.001
**IVRT, msec**	113.96 ± 12.15	95.26 ± 15.99	0.022
**E/A (-)**	1.77 ± 0.33	1.89 ± 0.51	0.355
**LVEDD, mm**	48.80 ± 3.99	31.40 ± 6.31	< 0.001
**MPI (-)**	0.55 ± 0.11	0.40 ± 0.06	< 0.001

^a^ Data are presented in mean ± SD.

^b^ Abbreviations: EF, ejection fraction; FS, fractional shorting; LVM, left ventricular mass; LVMI, left ventricular mass index; IVRT, isovolumic relaxation time; E/A, peak E velocity to peak A velocity ratio; LVEDD, left ventricular end-diastolic dimension; and MPI, myocardial performance index.

The mean of QT in the case and control groups were 348.95 ± 41.13 and 344.03 ± 31.56 msec, respectively (P = 0.030). The mean of QTc in cases and controls were 416.50 ± 16.24 and 392.03 ± 30.75 msec, respectively (P = 0.001). The mean of QTd in cases and controls were 66.26 ± 17.97 and 31.83 ± 9.11 msec, respectively (P = 0.001). The mean of QTcD in the case and control groups were 77.21 ± 18.35 and 38.24 ± 8.29 msec, respectively (P = 0.001). The mean of S wave in V_1_ in cases and controls were 8.22 ± 1.98 and 7.03 ± 3.21 mV, respectively (P = 0.011). The mean of R wave in V_5_ in the cases and controls were 18.04 ± 4.14 and 13.37 ± 5.02 mV, respectively (P = 0.001). All of the above data are shown in Table 3.

**Table 3.** Electrocardiographic Parameters in Case and Control Groups ^a, b^

Parameter	Patients	Controls	P value
**QT, msec**	348.95 ± 41.13	344.03 ± 31.56	0.030
**QT d, msec**	66.26 ± 17.97	31.83 ± 9.11	< 0.001
**QT c, msec**	416.50 ± 16.24	392.03 ± 30.75	< 0.001
**QTcd, msec**	77.21 ± 18.35	38.24 ± 8.29	< 0.001
**S in V **_**1**_ **, mV**	8.22 ± 1.98	7.03 ± 3.21	0.011
**R inV **_**5**_ **, mV**	18.04 ± 4.14	13.37 ± 5.02	< 0.001

^a^ Abbreviations: QTc, corrected QT ; QTcd, corrected QT dispersion; QTd, QT dispersion.

^b^ Data are presented in mean ± SD.

**Table 4.** Positive Predicted Value, Negative Predicted Value, Negative Likelihood Ratio, Positive Likelihood Ratio, and Youden's Index ^a, b^

Parameter	Positive Predicted Value	Negative Predicted Value	Negative Likelihood Ratio	Positive Likelihood Ratio	Youden's Index		
					Value	Sensitivity	Specificity
**LVM**	75.7	86.0	0.15	3.86	0.60	0.88	0.72
**LVMI**	81.3	85.7	0.17	4.34	0.77	0.80	0.97
**QT c**	64.1	66.1	0.51	1.78	0.367	0.87	0.50
**QT d**	80.0	92.0	0.09	4.00	0.82	0.88	0.93
**QTcd**	87.3	91.2	0.10	6.89	0.85	0.87	0.98

^a^ Abbreviations: LVM, left ventricular mass; LVMI, left ventricular mass index; QTc, corrected QT; QTd, QT dispersion; QTcd, corrected QT dispersion.

^b^ Youden’s index values were 0.6 for LVM (sensitivity = 0.88, specificity = 0.72), 0.77 for LVMI (sensitivity = 0.8, specificity = 0.97), 0.367 for QTc (sensitivity = 0. 87, specificity = 0.5), 0.82 for QTd (sensitivity = 0.88, specificity = 0.93), and 0.85 for QTcd (sensitivity = 0.87, specificity = 0.98).

**Figure 1.**
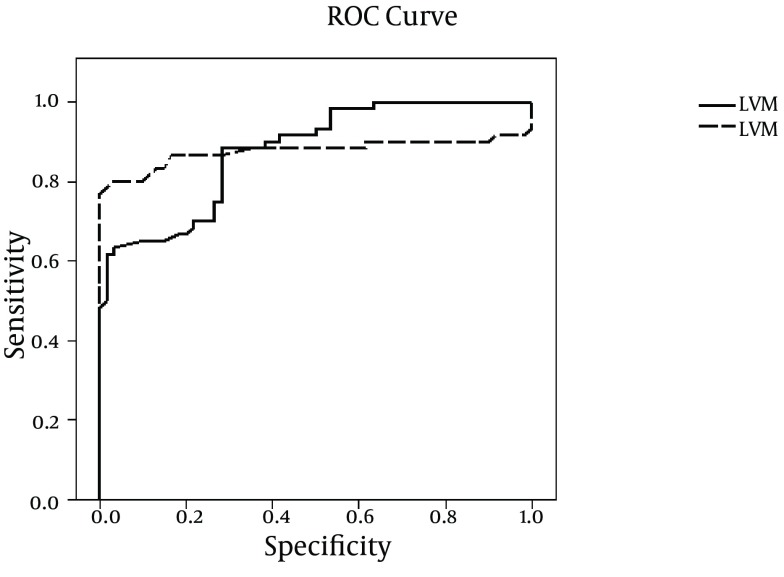
Sensitivity and Specificity of Left Ventricular Mass and Left Ventricular Mass Index

The sensitivity and specificity of LVM with cut-off point of 77.6 were 88.3 and 77.1, respectively. The sensitivity and specificity of LVMI with cut-off point of 27.1 were 86.7 and 80, respectively (Figure 1).

**Figure 2.**
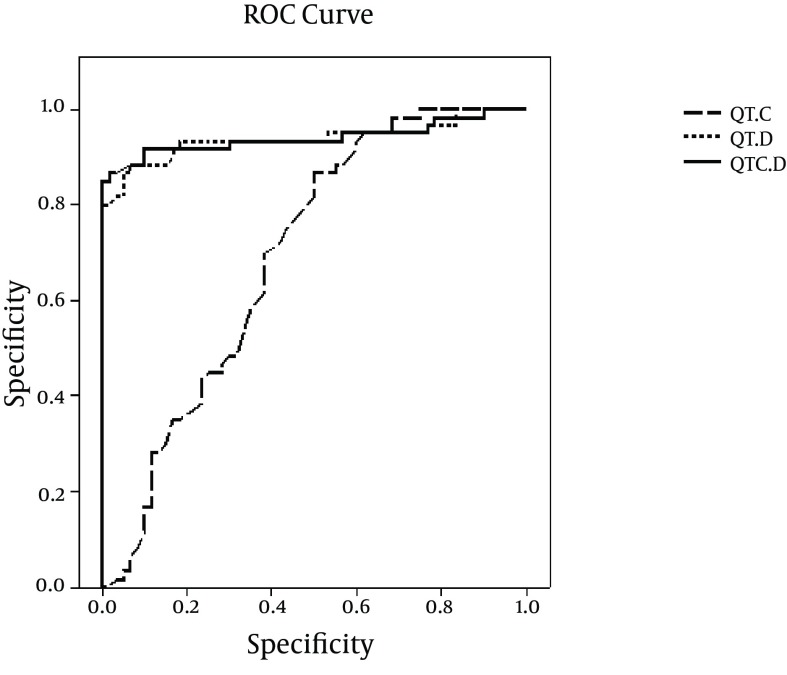
Sensitivity and Specificity of Corrected QT, QT Dispersion, and Corrected QT Dispersion

The sensitivity and specificity of QTc with cut-off point of 408.5 were 68.3 and 61.7, respectively. The sensitivity and specificity of QTd with cut-off point of 37 were 93.3 and 80. The Sensitivity and specificity of QTcd with cut-off point of 49.25 were 91.7 and 86.7, respectively (Figure 2).

## 5. Discussion

In the present study, 60 asymptomatic patients with thalassemia major and 60 healthy individuals older than ten years of age were evaluated by simultaneous standard two-dimensional echocardiography and ECG. We compared diagnostic values of QTcd and QTd with LVMI in these patients for early detection of cardiac dysfunction. Mean heart rate in patients with thalassemia was 92.28 ± 10.6 beats per minute, which was above the normal range (according to the previous studies). This result confirms the previous results by Noori et al. in 2007 that showed an association between tachycardia in patients with thalassemia in comparison with healthy individuals (13).

The mean of FS and SF in the case group were 65.9 ± 6.6 and 37.61 ± 4.9, respectively, which were in the normal range. It might be mainly caused by the fact that the patients who had severe reduction of EF and FS might have had symptoms of heart failure; hence, they were excluded from the study. The mean LVM was 102.51 ± 21.1 and when corrected to the height of participants, it was expressed as LVMI (g/m^2.7^). Mean of LVMI was 35.48 ± 7.8 in the case group, which was in agreement with results by Ulger (6).

To assess the sensitivity and specificity of QTd and QTcdversus LVMI, which was the standard method in the present study to detect probable and asymptomatic heart failure, we needed a cut-off point for LVMI such as the maximum LVMI in healthy participants. As a result, we used the cut-off point of 1.27. It is worth noting that the LVMI cannot definitely establish the presence of cardiac dysfunction in asymptomatic patients with thalassemia and patients with normal LVMI might have some degrees of heart failure. However, this degree of dysfunction is in the cellular level and was beyond the scope of the present investigation.

The main purpose of our study was to find the association of QTd and QTcD with LVMI as well as to clarify the diagnostic value of these indices for early detection of cardiac dysfunction. In a study in 2006, Ulger et al. showed the presence of this association (6) that was in agreement with our results; however, our results had less scattering that revealed a stronger association between them. Moreover, diagnostic values of electrocardiographic indices were not investigated in the aforementioned study. Our study showed that the sensitivity and specificity of LVMI in detecting cardiac dysfunction were 86.7% and 80%, respectively. QTc had a sensitivity and specificity of 68.3% and 61.7%, respectively. The sensitivity and specificity QTd were 93.3% and 80%, respectively. QTcd had a sensitivity and specificity of 91.7% and 86.7%, respectively. According to this information, there was no significant difference between LVMI and QTcd in terms of specificity and when the echocardiographic examination is not available, the electrocardiographic indices can be used as an alternative diagnostic test for early detection of cardiac involvement in patients with thalassemia major.

Therefore, QTcd is a more reliable indicator of cardiac dysfunction and impaired LVMI. Given the acceptable sensitivity and specificity of QTd and especially QTcd in comparison to echocardiography-derived LVMI in patients with thalassemia who do not have cardiac symptoms, we can use these indices at low cost. If these indices were abnormal, the patient should be referred for more accurate assessment of heart failure, including a comprehensive echocardiographic examination.

Ulger et al. studied 62 asymptomatic patients with thalassemia major who received frequent blood transfusion and iron chelators, and 52 healthy individuals older than ten years of age (6). They explained the association between LVMI and QTcd and found that QTcd and LVMI not only were increased in patients with thalassemia major as compared to the control group, but also were associated with each other.

In the other study conducted by Koucharian et al. on asymptomatic patients with thalassemia who were receiving regular blood transfusions and iron chelators, higher QTcd was found in asymptomatic patients with thalassemia major than in healthy subjects (7).

 Another investigation by Kayrak et al. showed that repolarization parameters were prolonged and impaired in asymptomatic patients with major β-thalassemia in comparison to controls. However, they did not find any association between ECG findings and cardiac iron load in these patients (14). The study of Sayed et al. showed that QTc and QTd increased in patients with major beta thalassemia (15). They suggested that with considering the simplicity of ECG, QTd criteria would be helpful and valuable parameter in earlier detection of cardiac abnormalities in patients with thalassemia major. Their results were partially supported by the results of the present study. 

Magri et al. studied 30 asymptomatic patients with thalassemia major who were older than ten years of age (16). They calculated the LVMI 48 hours before blood transfusion and found that even in the early and asymptomatic stages of cardiomyopathy, there were significant differences between indices of ventricular myocardial function, especially LVMI, between cases and controls.

In another study on 38 patients with thalassemia major who were receiving regular blood transfusions and iron chelators, Garadah et al. found that changes in QT, especially in QTd, and ventricular filling were higher in patients than in healthy individuals (17). In addition, the patients in the thalassemia group were in lower reference percentiles of weight and height and they had higher heart rate in comparison with healthy children; their result was consistent with our findings.

These findings reflected the fact that although slight changes in myocardium (caused by any reason, probably iron deposition) had no clinical symptoms, they could affect systolic and diastolic echocardiographic parameters. In addition, the myocardial changes caused a significant increase in QTd in patients with thalassemia major. Although the increases in QTd could be found in other diseases that could have some effects on ventricular repolarization, none of the patients had diabetes, hypertension, chronic renal failure, and obesity. Increased QTd in these patients was secondary to increased ventricular heterogeneity (17).

Zareba et al. explained the effect of the ventricular repolarization changes on increased risk of arrhythmias and sudden cardiac death (18). According to this study, beat to beat changes in the QT is secondary to changes in myocardial ion channel activity, which is in part due to myocardial pathology of various etiologies including cardiomyopathy. Moreover, Zareba and his associates studied the effects of QT changes on outcome of patients with myocardial infarction-induced cardiomyopathy (18). Patients in our study had also significant changes in QT. Furthermore, in comparison with LVMI, these changes had equivalent sensitivity and specificity to QTcd.

In another study by Piccirillo et al. which assessed the QTd changes in patients with echocardiography-detected dilated cardiomyopathy who had no clinical signs of CHF, QTd was increased in cardiomyopathy and associated with sudden death and arrhythmias in these patients. The cause of this increase in QTd was altered repolarization heterogeneity secondary to the involvement of the ventricular myocardium ion channels. In the aforementioned study, as in our study, QTd was increased. However, their study was conducted on patients with dilated cardiomyopathy (19).

In accordance with our results, in a study by Russo et al. on patients with thalassemia and a mean age of 27 years, a significantly higher QTcD was reported in patients with thalassemia than in the control group (20). However, the sensitivity and specificity of QTcD to predict the risk of sudden death were both reported as 70% in their study, which is not consistent with our findings.

Wu et al. showed that increased iron deposition in the heart muscle tissues caused heterogeneity of myocardium that made it susceptible to arrhythmias and QT prolongation (21). Moreover, their study showed that long-term peritoneal dialysis was associated with increased QTc (21). 

Finally, it can be concluded that given the availability of the ECG, its independency to specialized personnel, and with respect to acceptable sensitivity and specificity of QTcD and QTd criteria in comparison to LVMI, we can use ECG criteria rather than the LVMI for early detection of cardiac involvement in patients with thalassemia major especially in asymptomatic patients.
